# Is Rituximab-Associated Hypogammaglobulinemia Always Linked to B-Cell Depletion?

**DOI:** 10.3390/children9020295

**Published:** 2022-02-21

**Authors:** Anthie Damianaki, Marianna Tzanoudaki, Maria Kanariou, Emmanouil Liatsis, Alexandros Panos, Alexandra Soldatou, Lydia Kossiva

**Affiliations:** 1 Second Department of Pediatrics, National and Kapodistrian University of Athens, “P & A Kyriakou” Children’s Hospital, 11527 Athens, Greece; alxpanos@gmail.com (A.P.); alex_soldatou@hotmail.com (A.S.); lydiakossiva@hotmail.com (L.K.); 2 Department of Immunology-Histocompatibility, “Aghia Sophia” Children’s Hospital, 11527 Athens, Greece; emainasgr@yahoo.gr (M.T.); manolisliatsis@gmail.com (E.L.); 3 Division of Pediatric Immunology, IASO Children’s Hospital, 11527 Athens, Greece; m.kanariou@gmail.com

**Keywords:** autoimmune hemolytic anemia, warm antibody, hypogammaglobulinemia, rituximab

## Abstract

We describe a case of a 3-year-old male toddler with a history of severe and refractory warm antibody autoimmune hemolytic anemia (w-AIHA) since early infancy and hypogammaglobulinemia persisting 20 months after rituximab administration (second-line rescue therapy). Specifically, although peripheral blood flow cytometry B-cell population counts signified B-cell recovery following completion of rituximab therapy, IgG levels were barely detectable. Detailed laboratory evaluation did not reveal any humoral or cell-mediated immunity impairment and the patient remained asymptomatic, without any infections or recurrence of w-AIHA. Due to severe hypogammaglobulinemia, he was placed on immunoglobulin replacement therapy (IVIG). The implemented PID (primary immunodeficiency) gene panel identified only variants of uncertain significance (VUS). The aim of this report is to underline the documentation of persisting hypogammaglobulinemia after rituximab despite peripheral blood B-cell reconstitution.

## 1. Introduction

The incidence of antibody autoimmune hemolytic anemia (AIHA) is 1–3/100,000 people/year [1]. W-AIHA is the most common type, comprising approximately 70% of adult and 50% of pediatric cases [2]. The first-line treatment of w-AIHA includes IVIG and corticosteroids. Transfusion with packed red blood cells (PRBC) is indicated only in very severe hemolysis [2]. In children, AIHA often presents acutely, is self-limited and responds well to first-line treatment in the majority of cases [3]. Rarely, AIHA can be extremely severe in patients with a chronic course of the disease [4]. Especially in children younger than 2 years, the clinical course of the disease can show either resistance to intravenous immunoglobulin (IVIG) or dependence on high-dose steroids necessitating the use of second-line treatment [5].

Rituximab, a genetically engineered chimeric, murine/human IgG1/k monoclonal antibody specific for the CD20 antigen, is proposed as a second-line therapeutic option. Targeting the cells responsible for autoantibody production, it induces rapid in vivo depletion of B lymphocytes [5]. Rituximab was FDA-approved only for non-Hodgkin’s lymphoma (NHL), granulomatosis with polyangiitis (GPA) and microscopic polyangiitis (MPA) in children older than 2 years in combination with steroids. Its use as an off-label drug for the treatment for autoimmune cytopenias in immunocompetent children has been well described and has demonstrated an acceptable safety profile [6,7].

Rituximab can lead to immunosuppression through B-cell depletion causing hypogammaglobulinemia. Hypogammaglobulinemia is expected to be transient in people with previously normal immunoglobulin levels since both plasma cells and pro-B-cells lack CD20 expression [8,9]. In children with autoimmune cytopenias treated with rituximab, peripheral B-cell depletion occurs within two weeks after the first dose and generally persists for 2–12 months [4,7]. Although there are reports of prolonged depletion, they concern mostly high-risk patients, affected by malignant or autoimmune disorders [7]. In children, existing data on recovery of B-cells and IgG levels are limited to case series [10]. Recent reports underline that up to 30–50% of children exhibited transiently or persistently low IgG levels following rituximab despite normal pre-existing IgG levels [11]. Persistent hypogammaglobulinemia (PH) requiring immunoglobulin replacement has been described in children with autoimmune conditions treated with rituximab who were finally diagnosed with a primary immunodeficiency (PID). Therefore, rituximab-induced B-cell perturbation may unveil a primary intrinsic defect of the immune system [12]. In these cases, post-rituximab hypogammaglobulinemia has always been associated with delayed B-cell recovery. To the best of our knowledge, this is the first report of an asymptomatic pediatric patient with persistent hypogammaglobulinemia after rituximab despite B-cell reconstitution without an identified inborn error of immunity.

## 2. Case Presentation

The case we describe here is that of a 3-year-old male toddler with a history of a severe and refractory w-AIHA from early infancy who presented with persisting hypogammaglobulinemia for more than 20 months after the last dose of rituximab, which had been administrated as second-line rescue therapy.

He is the second child of non-consanguineous parents, born as full-term with an unremarkable antenatal history. There was no maternal history of miscarriages. A strong family history of autoimmune disorders was noted, including his mother with Hashimoto thyroiditis, his father with gyroid alopecia and his paternal grand-father with localized scleroderma.

The patient was diagnosed with severe AIHA at the age of 2.5 months characterized by warm autoantibodies activating complement (panagglutinins). Complete blood count (CBC) on admission was as follows: HB: 4.4 g/dL; hematocrit (HCT): 12.7%; reticulocyte count (REC): 2%; mean corpuscular volume (MCV): 81.4 fL; mean corpuscular hemoglobin (MCH): 28.2 pg; red cell distribution width (RDW): 16.5; white blood cell (WBC): 14,700 × 10^3^/μL (neutrophils: 15%; lymphocytes: 80%; monocytes: 3.3%); platelets: 522,000 × 10^3^/μL. REC was not elevated early in the course but anisocytosis, polychromasia and poikilocytosis were noted on peripheral blood smear, indicating reticulocytosis. The direct agglutination test (DAT) (IgG^4+^, C3d^2+^) and indirect agglutination test (IAT) were both positive. No infection or systemic disease at the time of diagnosis was found. There were no indications of a preceding or concurrent infection at the time of diagnosis. The infant had received his first hexavalent vaccine 15 days earlier.

Due to recurrent episodes of w-AIHA, the patient was hospitalized 5 times until the age of 15 months, receiving PRBC transfusions, 3 courses of IVIG (1 g/kg/course) and high doses of intravenous corticosteroids 4 times. The first relapse occurred at the age of 5 months at a regular follow-up visit 40 days after the steroid therapy cessation; then he received a PRBC transfusion and one course of IVIG. Hb was stabilized at 10.8 gr/dL. The second relapse took place in the seventh month of age and the infant was started again on high corticosteroid oral therapy. The third relapse followed 7 months later at the age of 15 months, despite ongoing steroid therapy for 8 months. Between the second and the third relapse, Hb levels ranged from 7.5 to 10 g/dL. The third relapse occurred in the context of roseola infantum. During this hospitalization, the hemolysis was not controlled, transfusion-dependence persisted, large amounts of steroids were administrated intravenously on top and second-line treatment with rituximab was started after the permission of the National Drug Administration Committee. The patient received four weekly doses (375 mg/m^2^) with no infusion-related side effects. Seven days after the fourth dose, he achieved adequate hematological response, but DAT was still positive. The patient continued steroid therapy on a weaning dose for 3 months and no relapse of hemolysis has been noted ever since.

Due to the severe and refractory presentation of w-AIHA in this patient, a thorough workup was conducted. The patient was seronegative for HIV-1 and HIV-2 and other infections (EBV, CMV, HSV, toxoplasma, rubella, parvovirus B-19, influenza virus). Hematological malignancies were ruled out by appropriate diagnostic procedures. Initial immunoglobulin levels measurement at the age of 2.5 months, prior to any therapeutic intervention, revealed IgM levels slightly below the age-adjusted normal range (25 mg/dL, normal range 31–135). All the complement fragments (C3, C4, C1q, C2, C5, C6, C7, C8, C9) were within normal range. Twice 50% complement hemolytic activity (CH50) was measured and was found to be slightly lower than normal (63%; normal range: 69–129%), possibly due to the fact that all the CH50 measurements were performed during the hemolytic crisis. C1 esterase inhibitor activity and quantification measurement was normal, as well as antinuclear antibody (AΝA), anti-DNA and a broad range of AuAbs for autoimmune systematic diseases which were all checked twice and found to be normal.

The current concerning issue is the prolonged hypogammaglobulinemia that is constantly present 20 months after the rituximab therapy completion. As demonstrated in Table 1, which compares the immunophenotype of T- and B-cells in peripheral blood before and after rituximab therapy, peripheral blood lymphocyte count flow cytometry was also normal at the age of 10 months, while steroids had been received for 3 months. Peripheral blood flow cytometry for B-cells demonstrated that B-cell memory subsets, especially the CD27+IgM-IgD- switched memory (sm) B-cells, were within normal range. The percentages of CD21+(low)CD38+ B-cells and CD4-CD8-TCRαβ+ Τ-lymphocytes were also low.

**Figure 1:**
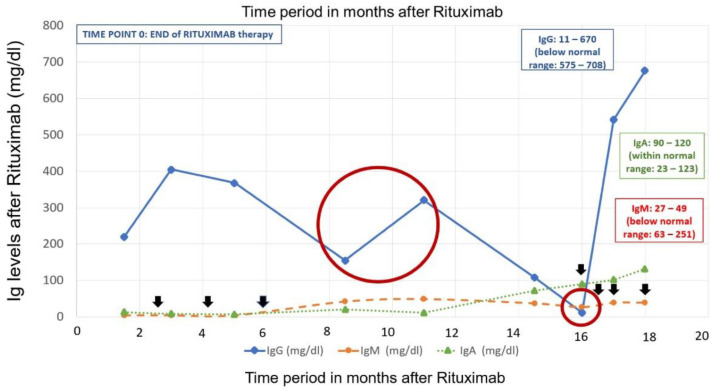
Levels of immunoglobulins after rituximab therapymin: minimum; max: maximum; IgA/IgG/IgM: immunogIobulin A/G/M. Black arrows indicate the IVIG (intravenous immunoglobulin) administration time periods. Time period 0 signifies the end of rituximab therapy. The large red circle emphasizes the IgG/IgM double increase during the vaccination period, while off IVIG replacement therapy for 5 months. The small red circle indicates the time point (16 months after rituximab) of the IgG/IgM nadir despite B-cell reconstitution in the peripheral blood. At that point, vaccine antibody levels were measured and found subnormal. IgA levels normalized approximately 14 months after rituximab therapy.

Prior to rituximab therapy, 11 months after the last IVIG dose and while on steroids for 8 months, peripheral blood flow cytometry for B-cells was performed and found to be normal again. The immunoglobulin levels were as follows: IgG 486 mg/dL (range 575–1446), IgM 52 (range 63–251), IgA 40 (range 23–123). During rituximab therapy, B-cells became undetectable and IgG levels gradually decreased. Although he did not suffer from any infections, he commenced on regular IVIG (0.4 g/kg/dose) replacement therapy for three consecutive months due to sustained low IgG levels. Afterwards, there was an 11-month free of IVIG replacement period when the patient was evaluated by his private physician due to the COVID-19 pandemic. During this time, the patient was asymptomatic, while his immunoglobulins were decreasing. IgG reached a nadir level of 11 mg/dL (Table 2, Figure 1). By that time, it was 16 months from the last rituximab dose, 11 months from the last IVIG course and 14 months off steroid therapy. IgM was also low, while IgA had already been normalized. Surprisingly, peripheral blood flow cytometry for B-cells was rechecked, and B-cell memory subsets were again normal. There was no sign of B-cell depletion in the peripheral blood that could explain the IgG/IgM deficiency. Nevertheless, it is worth mentioning that before reaching nadir, both IgG and IgM were elevated through the preceding months (from 155 to 320 mg/dL and from 20 to 49 mg/dL, respectively). We speculate that this could be explained by a scheduled vaccination with pneumococcal conjugate vaccine and vaccination against tetanus and *Haemophilus influenzae* type B antigens.

Regarding the patient’s medical history, he has no history of infections, no failure to thrive and his clinical examination was unremarkable through all these years. The CBC and biochemical screening were also within normal range. Due to severe hypogammaglobulinemia, he was placed on regular IVIG replacement therapy to maintain IgG above 500 mg/dL. His Hb was stable (>12 gr/dL with 1–2% REC) for the last 18 months, but DAT was still positive (IgA: +/−, IgM: 2+, C3d: 2+) without signs of active hemolysis. While off IVIG, antibodies against pneumococcus, tetanus and HIB antigens were measured to be subnormal. The isohemagglutinins were present, complement fragments were normal again, while IgG3 remained below 2 SD for age-related normal range.

In conclusion, considering the long-term history of the patient and the unrevealing immunological workup, a PID panel of 407 genes was implemented free of charge in order to identify an underlying intrinsic defect of the immune system. The gene panel revealed only variants of uncertain significance. There has been no evidence of pathogenicity of these variants until now and the clinical significance of the variants identified in these genes is uncertain according to the dbSNP and ExAc source database (Table 3).

## 3. Discussion

We reported on an infant with severe and refractory AIHA since the first months of life, necessitating off-label use of rituximab and ensuing extremely low immunoglobulin levels 20 months after treatment. The first question that arises from this report is whether this case demonstrates a profound rituximab-induced hypogammaglobulinemia or an underlying PID unmasked by rituximab. In addition, most post-rituximab PH is a frequent condition in children with autoimmune cytopenia. Patients with post-rituximab PH are eventually diagnosed with a PID, more frequently common variable immunodeficiency (CVID) or autoimmune lymphoproliferative syndrome (ALPS) [11,12,13].

M.M.G Adeli et al. recently characterized this phenomenon as persistent antibody depletion after rituximab (PADAR), occurring after the treatment of autoimmune cytopenias with rituximab, and posed the question of whether PADAR may have been a consequence of the rituximab therapy or the development of CVID as part of the natural history of the underlying autoimmune cytopenias. Indeed, autoimmune cytopenias may be the initial manifestation of CVID [9]. The study of Khojah et al. suggested that pediatric patients are more susceptible to rituximab-associated hypogammaglobulinemia than adults, perhaps due to immaturity of the immune system with a lower percentage of memory B-cells [14,15].

Taking into consideration the medical history of our patient, who remained free of infections of the respiratory and gastrointestinal tracts which are characteristic of impaired humoral immunity. He is tracking along the 50th percentile of height and weight on the growth chart and has no clinical sign or imaging findings of an immunodeficiency including lymphadenopathy, organomegaly, granulomatosis or recurrent infections [16] Other red flags from the clinical history of our patient, such as overreaction in vaccines, are absent. We could speculate that the very first vaccine, received at the age of 2 months, may have triggered the onset of w-AIHA, since no other precipitating cause could be found. In addition, cases of AIHA have been described after immunization with active varicella vaccines [17]. Finally, a family history of PID is not documented in our patient. Although both the clinical and the laboratory profile of our patient do not point towards a PID, we certainly cannot exclude the possibility that our patient’s clinical manifestations could be due to an underlying monogenic inborn error of immunity that cannot be established through the current diagnostic procedures.

Regarding the immunological workup, peripheral blood flow cytometry evaluation was normal. The percentage of CD21lowCD38low B-cells was low. This B-cell population has been characterized as tissue homing, innate-like B-cells, containing autoreactive unresponsive B-cell clones, which are expanded in autoimmune diseases and immunodeficiencies. Several autoimmune diseases are characterized by an expansion of plasmablasts/plasma cells in the peripheral blood, indicating aberrant B-cell activation; frequencies of plasmablasts never exceeded 5% of total B-cells. An expansion of transitional B-cells in diverse patient populations reconstituting after B-cell depletion therapy due to rituximab, including lymphomas and autoimmune diseases, has also been reported in the literature [18]. Finally, considering the toxicity of rituximab in ALPS patients, causing prolonged hypogammaglobulinemia up to life-long IVIG dependency, the percentage of CD4-CD8-TCRαβ+ Τ-lymphocytes was low [12,14].

It has been suggested that decreased numbers of memory B-cells might be an indicator of a subgroup of CVID patients bearing an increased risk for severe prognosis. Interestingly, in all pediatric cases describing rituximab-associated hypogammaglobulinemia, low level immunoglobulins were strictly associated with prolonged B-cell depletion. According to Ottaviano et al., even some patients with persistent hypogammaglobulinemia with normal B-cells required long-term IVIG replacement therapy, but these children could present an intrinsic defect in (sm) B-cell maturation or hypogammaglobulinemia that could be the result of an impaired cognate T-helper (TH)–B-cell interaction [11,19]. None of the aforementioned factors were observed in our patient workup. The paradox here is that peripheral blood flow cytometry for B-cells was indicative of B-cell reconstitution, while IgG was almost undetectable. This is the first time that this has been reported.

In our patient’s immunological workup, IgG3 level was recorded as below 2 SD for age, twice, whereas IgA levels were within normal range. We hypothesize that the low IgG3 level is clinically insignificant as long as he remains free from infections and that it can be explained by the maturational delay often observed in young children. IgG3 deficiency, in particular, has a relatively good chance of normalizing by up to 6 years of age [20,21]. Additionally, IgM level is always slightly below the lowest normal range, although not always less than 2 SD for age. IgG was also slightly below normal at the age of 15 months prior to starting rituximab therapy, but considering that this measurement was conducted while on steroids for 8 months, the IgG level was not so unexpected according to expert opinion [21,22].

It is not crystal clear what is the role of rituximab therapy in our case as far as prolonged immunosuppression is concerned. While true PID unmasked by rituximab in our patient is an emerging concern, longer observation and clinical follow-up will clarify if rituximab does potentiate underlying immune dysregulation in this patient or if rituximab alone has the potential to cause PADAR in the absence of an underlying immune dysregulation due to B-cell perturbation that could unbalance T- and B-lymphocyte homeostasis, resulting in ineffective immunoglobulin production. The fact that a scheduled vaccination probably led to an unexpected increase of IgG/IgM before reaching nadir underlines the possibility that the effect of rituximab on this prolonged hypogammaglobulinemia is probably not supported and this hypogammaglobulinemia seems to be a manifestation of an intrinsic defect of the Immune system [23].

Unfortunately, the genetic testing revealed mostly variants that are not yet present in a population database and which represent genetic changes whose impact on the individual’s clinical history is not yet known. Although all of them codify sequence changes that replace one amino acid with another, these changes are characterized as missense and do not adversely affect protein function; thus, their pathogenic role is unclear. Finally, they do not correspond with the patient’s medical and family history; for example, sequence changes of the COL7A1 or the TERT genes are associated with epidermolysis bullosa or dyskeratosis congenita, respectively.

## 4. Conclusions

PH after rituximab therapy for autoimmune cytopenias can occur despite B-cell reconstitution, as demonstrated by an adequate number of B-cells in peripheral blood flow cytometry. In this case, there are numerous controversies over whether PH could be interpreted either as an iatrogenic immunological impairment or as an underlying intrinsic defect of the immune system provoked by rituximab. A targeted diagnostic workup including a gene panel might be an enlightening diagnostic tool for identifying novel pathogenic gene variants linked to PID. Although the genetic testing may identify only VUS, as in our patient, the classification of variants may change over time as a result of new variant interpretation guidelines and/or new information. In addition, the limited number of genes detected by the PID gene panel in our patient would detect the implementation of another more extended gene panel (NGS, WES) to establish the PID diagnosis. Therefore, the scenario of an emerging PID diagnosis in our patient cannot be denied and so clinical follow up is warranted and vigilant monitoring will be required.

## Figures and Tables

**Table 1:** Peripheral blood T- and B-cell immunophenotypes before and after RITUXIMAB therapy. Concurrent immunoglobulin levels are demonstrated.

	Age: 10 Months Old	Age: 33 Months Old
	RITUXIMAB	
	6 months before RITUXIMAB therapy	16 months after RITUXIMAB therapy
	Current therapy	
**Immunophenotype of T & B cells in the peripheral blood**	Oral steroid therapy for 3 months ^1^	None ^2^
**Lymphocytes/µl**	3997 (2600–10,400)	5821 (1700–6900)
**CD3+ (% on Lymphs)**	63.8% (54–76)	68% (43–76)
**CD3+/µl**	2549 (1600–6700)	3958 (900–4500)
**CD3+ CD4+ T helper% lymphs**	43.1% (31–54)	39.8% (23–48)
**CD3+ CD4+/µl**	1724 (1000–4600)	2320 (500–2400)
**CD3+ CD8+ T cytotoxic% lymphs**	19.6% (12–28)	23.2% (14–33)
**CD3+ CD8+/µl**	781 (400–2100)	1348 (300–1600)
**CD3+ CD4+/CD3+ CD8+**	2.2 (1.3–3.9)	1.7 (0.9–2.9)
**CD19+ Bcells% lymphs**	21.4% (15–39)	22.5% (14–44)
**CD19+ Bcells/µl**	837 (600–2700)	1557 (200–2100)
**CD19+ CD20+ mature B on lymphs**	19.7%	22.2%
**CD19+ CD20+/µl**	771	1537
**CD3-CD16/56+ NKcells% on lymphs**	16% (3–17)	8.6% (4–23)
**CD3-CD16/56+ NKcells/µl**	608 (200–1200)	602 (100–1400)
**CD3+ HLADR+ Activated T% on CD3**	1.7% (2–8)	2% (1.5–7)
**CD3+ TCR γδ+% on CD3+**	1.4% (2–15)	8.1% (4–25)
**CD4+ CD45RA+ naive on CD4+**	86.2% (64–93)	80.8% (53–86)
**CD27- naive% on B cells**	76.5% (74.7–90.5)	90.9% (77–90)
**IgD+ CD27+% on B cells**	11.5% (4.9–14.2)	5.5% (5–14)
**IgD-CD27+% on B cells**	9.2% (2.9–2.2)	3.5% (3–8)
**CD21lowCD38-% on B cells**	4.5% (<10)	5.2% (<10)
**Immunoglobulin levels (mg/dl)**		
**IgG**	688 (316–1148)	11 (708–1622)
**IgM**	37 (47–204)	27 (69–251)
**IgA**	60 (13–69)	90 (32–245)

IgA/IgG/IgM: immunoglobulin A/G/M. Figures in bold indicate abnormal values. ^1^ Last IVIG course (1 g/kg/dose) 5 months prior. ^2^ Last IVIG replacement therapy (0.4 g/kg/dose) 11 months prior. ^2^ Steroid therapy cessation 14 months prior. Age-matched normal values in brackets.

**Table 2:**
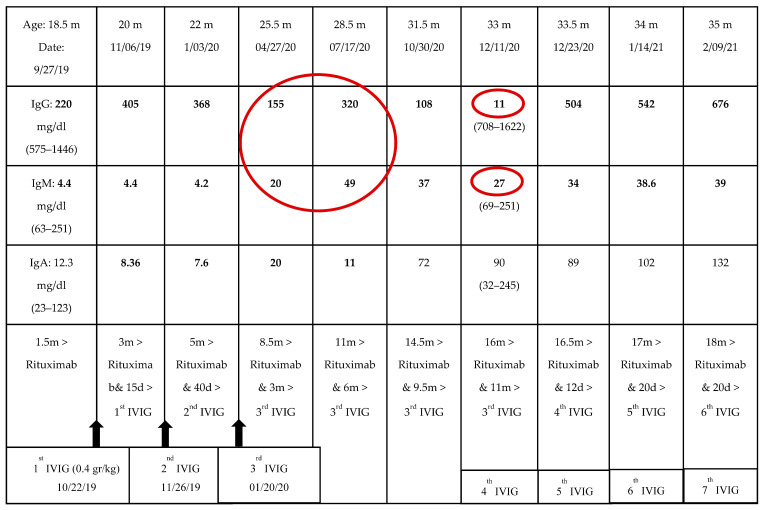
Levels of immunoglobulins after rituximab.

m: months; d: days; IgA/IgG/IgM: immunogIobulin A/G/M; IVIG: intravenous immunoglobulin. Black arrows indicate the IVIG administration time periods. Figures in bold indicate abnormal values. Greater-than sign “>” stands for “after”. The large red circle emphasizes the IgG/IgM double increase during the vaccination period, while off IVIG replacement therapy for 5 months. The small red circle indicates the time point (16 months after rituximab) of the IgG/IgM nadir despite B-cell reconstitution in the peripheral blood. At that point, vaccine antibody levels were measured and found subnormal.

**Table 3:** Diagnostic genetic testing resulted in the identification of variants of uncertain significance (VUS).

GENE	VARIANT	ZYGOSITY	VARIANT CLASSIFICATION
**C6**	c.820C > A (p.Gln274Lys)	Heterozygous	Uncertain Significance
**CIITA**	c.2320C > T (p.Leu774Phe)	Heterozygous	Uncertain Significance
**COL7A1**	c.2858-3C > T (Intronic)	Heterozygous	Uncertain Significance
**TERT**	c.2573G > A (p.Arg858Gln)	Heterozygous	Uncertain Significance
**TP63**	c.1537G > C (p.Ala513Pro)	Heterozygous	Uncertain Significance
**WDR1**	c.512C > T (p.Ala171Val)	Heterozygous	Uncertain Significance
**ZCCHC8**	c.1652G > A (p.Gly551Asp)	Heterozygous	Uncertain Significance

Example of interpretation for the second column: the sequence change “c.820C > A” replaces glutamine with lysine at codon 274 of the C6 protein (p.Gln274Lys).

## Data Availability

Population frequencies are derived from public sites that aggregate data from large-scale population sequencing projects including ExAC (http://exac.broadinstitute.org) and dbSNP (http://ncbi.nlm.nih.gov/SNP).

## Abbreviations

AIHA: Autoimmune Hemolytic Anemia; ALPS: Autoimmune Lymphoproliferative Syndrome; ANA: Antinuclear Antibody; AuAbs: AutoAntibodies; CBC: Complete Blood Count; CD20: Cluster of Differentiate 20; CH50: 50% Complement Hemolytic activity; CLL: Chronic Lymphoblastic Leukemia; CMV: Cytomegalovirus; CVID: Common Variable Immunodeficiency; DAT: Direct Agglutination Test; DNA: Deoxyribonucleic Acid; EBV: Epstein–Barr Virus; ENA: Extractable Nuclear Antigen; FDA: Food and Drug Administration; GPA: Granulomatosis with Polyangiitis; HB: Hemoglobin; HCT: Hematocrit; HIV: Human Immunodeficiency Virus; HSV: Herpes Simplex Virus; IAT: Indirect Agglutination Test; IgA/IgG/IgM: ImmunogIobulin A/G/M; IVIG: Intravenous Immunoglobulin; MCV: Mean Corpuscular Volume; MCH: Mean Corpuscular Hemoglobin; MPA: Microscopic Polyangiitis; NGS: Next Generation Sequencing; NHL: Non-Hodgkin’s Lymphoma; PH: Persistent Hypogammaglobulinemia; PID: Primary Immunodeficiency; PRBC: Packed Red Blood Cells; PNH: Paroxysmal Nocturnal Hemoglobinuria; RDW: Red Cell Distribution Width; REC: Reticulocyte Count; SLE: Systemic Lupus Erythematosus; SD: Standard Deviation; SM: Switched Memory; VUS: Variants of Uncertain Significance; w-AIHA: Warm Antibody Autoimmune Hemolytic Anemia; WBC: White Blood Cell; WES: Whole Exome Sequencing.

## References

[1] Michel M. (2011). Classification and therapeutic approaches in autoimmune hemolytic anemia: An update. Expert Rev. Hematol..

[2] Kalfa T.A. (2016). Warm antibody autoimmune hemolytic anemia. Hematology.

[3] Zecca M., Nobili B., Ramenghi U., Perrotta S., Amendola G., Rosito P., Jankovic M., Pierani P., De Stefano P., Bonora M.R. (2003). Rituximab for the treatment of refractory autoimmune hemolytic anemia in children. Blood.

[4] Quartier P., Brethon B., Philippet P., Landman-Parker J., Le Deist F., Fischer A. (2001). Treatment of childhood autoimmune haemolytic anaemia with rituximab. Lancet.

[5] Packman C.H. (2015). The Clinical Pictures of Autoimmune Hemolytic Anemia. Transfus. Med. Hemotherapy.

[6] Reff M.E., Carner K., Chambers S., Chinn P.C., Leonard J.E., Raab R., Newman R.A., Hanna N., Anderson D.R. (1994). Depletion of B-cells in vivo by a chimeric mouse monoclonal antibody to CD20. Blood.

[7] Bader-Meunier B., Aladjidi N., Bellmann F., Monpoux F., Nelken B., Robert A., Armari-Alla C., Picard C., Ledeist F., Munzer M. (2007). Rituximab therapy for childhood Evans syndrome. Haematologica.

[8] El-Hallak M., Binstadt B.A., Leichtner A.M., Bennett C.M., Neufeld E.J., Fuhlbrigge R.C., Zurakowski D., Sundel R.P. (2007). Clinical effects and safety of rituximab for treatment of refractory pediatric autoimmune diseases. J. Pediatr..

[9] Adeli M.M.G., Eichner B.H., Thornburg C., Williams L. (2009). Persistent antibody depletion after rituximab in three children with autoimmune cytopenias. Pediatr. Hematol. Oncol..

[10] Svahn J., Fioredda F., Calvillo M., Molinari A.C., Micalizzi C., Banov L., Schmidt M., Caprino D., Marinelli D., Gallisai D. (2009). Rituximab-based immunosuppression for autoimmune haemolytic anaemia in infants. Br. J. Haematol..

[11] Labrosse R., Barmettler S., Derfalvi B., Blincoe A., Cros G., Lacombe-Barrios J., Barsalou J., Yang N., Alrumayyan N., Sinclair J. (2021). Rituximab-induced hypogammaglobulinemia and infection risk in pediatric patients. J. Allergy Clin. Immunol..

[12] Rao V.K., Price S., Perkins K., Aldridge P., Tretler J., Davis J., Dale J.K., Gill F., Hartman K.R., Stork L.C. (2009). Use of Rituximab for Refractory Cytopenias Associated With Autoimmune Lymphoproliferative Syndrome (ALPS). Pediatric Blood Cancer.

[13] Barmettler S., Mei-Sing O., Farmer J.R., Choi H., Walter J. (2018). Association of Immunoglobulin Levels, Infectious Risk, and Mortality With Rituximab and Hypogammaglobulinemia. JAMA Netw. Open.

[14] Bucciol G., Moens L., Bosch B., Bossuyt X., Casanova J.-L., Puel A., Meyts I. (2019). Lessons learned from the study of human inborn errors of innate immunity. J. Allergy Clin. Immunol..

[15] Khojah A.M., Miller M.L., Klein-Gitelman M.S., Curran M.L., Hans V., Pachman L.M., Fuleihan R.L. (2019). Rituximab-associated Hypogammaglobulinemia in pediatric patients with autoimmune diseases. Pediatric Rheumatol..

[16] Tangye S.G., Al-Herz W., Bousfiha A. (2020). Human Inborn Errors of Immunity: 2019 Update on the Classification form the International Union of Immunological Societies Expert Committee. J. Clin. Immunol..

[17] Biakci Z., Bozkurt H.B., Olcay L. (2019). Three Cases of Autoimmune Hemolytic Anemia following Primary Varicella Infection and Vaccination: Possible Pathogenesis in the Context of Current Information. Ann. Hematol. Oncol..

[18] Cunningham-Rundles C. (2002). Hematologic complications of primary immune deficiencies. Blood Rev..

[19] Ottaviano G., Marinoni M., Graziani S., Sibson K., Barzaghi F., Bertolini P., Chini L., Corti P., Cancrini C., D’Alba I. (2020). Rituximab Unveils Hypogammaglobulinemia and Immunodeficiency in Children with Autoimmune Cytopenia. J. Allergy Clin. Immunol. Pract..

[20] Karaca N.E., Karadeniz C., Aksu G., Kutukculer N. (2009). Clinical and laboratory evaluation of periodically monitored Turkish children with IgG subclass deficiencies. Asian Pac. J. Allergy Immunol..

[21] Wahn V., Von Bernuth H. (2017). IgG subclass deficiencies in children: Facts and fiction. Pediatr. Allergy Immunol..

[22] McMillan R., Longmire R., Yelenosky R. (1976). The Effect of Corticosteroids on Human IgG Synthesis. J. Immunol..

[23] Siegrist C.-A. (2018). Vaccine Immunology. Plotkin’s Vaccines.

